# A Linear-Time Algorithm for 4-Coloring Some Classes of Planar Graphs

**DOI:** 10.1155/2021/7667656

**Published:** 2021-10-05

**Authors:** Zuosong Liang, Huandi Wei

**Affiliations:** ^1^School of Management, Qufu Normal University, Rizhao 276826, China; ^2^School of Library, Qufu Normal University, Rizhao 276826, China

## Abstract

Every graph *G*=(*V*, *E*) considered in this paper consists of a finite set *V* of vertices and a finite set *E* of edges, together with an incidence function that associates each edge *e* ∈ *E* of *G* with an unordered pair of vertices of *G* which are called the ends of the edge *e*. A graph is said to be a planar graph if it can be drawn in the plane so that its edges intersect only at their ends. A proper *k*-vertex-coloring of a graph *G*=(*V*, *E*) is a mapping *c* : *V*⟶*S* (*S* is a set of *k* colors) such that no two adjacent vertices are assigned the same colors. The famous Four Color Theorem states that a planar graph has a proper vertex-coloring with four colors. However, the current known proof for the Four Color Theorem is computer assisted. In addition, the correctness of the proof is still lengthy and complicated. In 2010, a simple *O*(*n*^2^) time algorithm was provided to 4-color a 3-colorable planar graph. In this paper, we give an improved linear-time algorithm to either output a proper 4-coloring of *G* or conclude that *G* is not 3-colorable when an arbitrary planar graph *G* is given. Using this algorithm, we can get the proper 4-colorings of 3-colorable planar graphs, planar graphs with maximum degree at most five, and claw-free planar graphs.

## 1. Introduction

Every *graphG*=(*V*, *E*) considered here consists of a finite set *V* of vertices and a finite set *E* of edges, together with an incidence function that associates each edge of *G* with an unordered pair of vertices of *G*. If {*u*, *v*} is the unordered pair of vertices corresponding to the edge *e* of *G*, then *e* is denoted by *e*=*uv* and *u* is said to be *adjacent* to *v*. In addition, if *e*=*uv*, *e* is said to be *incident* to *u* and *v* and *u* and *v* are called the *ends* of *e*. For the standard terminology not given here, we refer the reader to [1]. The number of vertices of *G* is called the *order* of *G*. For a vertex *v* ∈ *V*, the *open neighborhoodN*(*v*) of *v* is defined as the set of vertices adjacent to *v*. The *closed neighborhoodN*[*v*] of *v* is defined as *N*[*v*]=*N*(*v*) ∪ {*v*}. The *degree* of *v* is equal to |*N*(*v*)|, denoted by *d*_*G*_(*v*) or simply *d*(*v*). By *δ*(*G*) and Δ(*G*), we denote the *minimum degree* and the *maximum degree* of graph *G*, respectively. A *k-regular graphG* is a graph such that every vertex of *G* has the degree *k*. For a subset *S* ⊆ *V*, the induced subgraph, denoted by *G*[*S*], is the subgraph of *G* whose vertex set is *S* and whose edge set consists of all edges of *G* which have both ends in *S*. A complete graph is a simple graph in which any two vertices are adjacent. Let *K*_*n*_ denote the complete graph on *n* vertices. Usually, *K*_3_ is called a *triangle*. A *cycle* on three or more vertices is a simple graph whose vertices can be arranged in a cyclic sequence in such a way that two vertices are adjacent if they are consecutive in the sequence and are nonadjacent otherwise. A graph *G* is bipartite if its vertex set can be partitioned into two sets *V*_1_ and *V*_2_ so that every edge has one end in *V*_1_ and the other one in *V*_2_. Specially, if every vertex in *V*_1_ is adjacent to every vertex in *V*_2_, then *G* is called a complete bipartite graph. As usual, *K*_*m*,*n*_ denotes a complete bipartite graph with classes of cardinality *m* and *n*. The graph *K*_1,3_ is also called a *claw*. Given a graph *F*, a graph *G* is *F-free* if it does not contain *F* as an induced subgraph. In particular, a *K*_1,3_-free graph is *claw-free*. By starting with a disjoint union of two graphs *G* and *H* and adding edges joining every vertex of *G* to every vertex of *H*, one obtains the *join* of *G* and *H*, denoted by *G* ∨ *H*. The join *C*_*n*_ ∨ *K*_1_ of a cycle *C*_*n*_ and a single vertex is referred to as a *n-wheel* denoted by *W*_*n*_.

A graph is said to be a *planar graph* if it can be drawn in the plane so that its edges intersect only at their ends. Such a drawing is called a *planar embedding* of the graph. Any such particular embedding is called a *plane graph*. A proper *k-vertex-coloring*, or simply a *properk-coloring*, of a graph *G*=(*V*, *E*) is a mapping *c* : *V*⟶*S* (*S* is a set of *k* colors) such that no two adjacent vertices are assigned the same colors. In this paper, we refer to a proper coloring as a “coloring” and to a proper *k*-coloring as a “*k*-coloring.” The famous Four Color Theorem states that a planar graph has a proper coloring with four colors. The original proof of the Four Color Theorem by Appel and Haken [2] and Appel et al. [3] relies heavily on the computer for checking details involved in finding an unavoidable set and verifying that all configurations in that set are reducible. It employs no fewer than 487 discharging rules, resulting in a set of over 1400 unavoidable configurations. The more recent proof by Robertson et al. [4], although also dependent on the computer, is simpler in many ways. In their proof, thirty-two discharging rules are needed, generating a list of 633 unavoidable configurations.

In 2010, a simple *O*(*n*^2^) time algorithm is provided to 4-color a 3-colorable planar graph by Kawarabayashi and Ozeki [5]. In this paper, given a planar graph *G*, we design an improved linear-time algorithm to either output a 4-coloring of *G* or conclude that *G* is not 3-colorable. Using this algorithm, we can get the 4-colorings of 3-colorable planar graphs, planar graphs with maximum degree at most five, and claw-free planar graphs.

## 2. Proof and Algorithm

**Figure 1:**
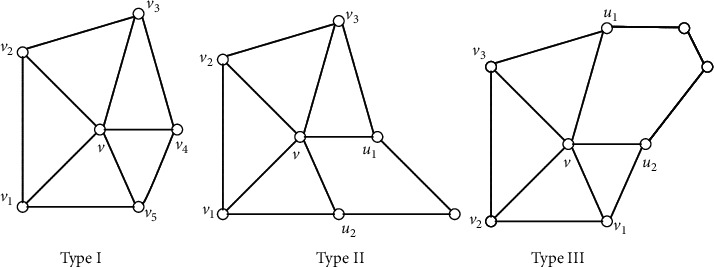
A bad vertex.

First, we give some definitions and lemmas which are related to our algorithm. A plane graph *G* partitions the plane into a number of arcwise-connected open sets which are called the *faces* of *G*. We call *f* a *k-face* if *f* is incident to *k* edges of *G*. For a simple plane graph *G*, we call a vertex *v* of degree five *bad* if all faces incident with *v*, except for the at most one, are triangles and the exceptional face has size at most five. Moreover, *v* is Type I, Type II, and Type III if the exceptional face is a triangle, a 4-face and a 5-face, respectively, as shown in Figure 1. Obviously, if a plane *G* has a bad vertex of Type I, then *G* is not 3-colorable. Note that, if *v* is a bad vertex of Type I of the plane graph, the induced subgraph *G*[*N*[*v*]] is not necessarily a 5-wheel since *G*[*N*(*v*)] may have a triangle and thus *G*[*N*[*v*]] has a *K*_4_. To *identify* nonadjacent vertices *x* and *y* of a graph *G* is to replace the two vertices by a single vertex incident to all the edges which are incident to either *x* or *y*. About the bad vertex *v* of Type II or III, the following observation is obvious.

**Observation 1 .**

Let *v* be a bad vertex of Type II or III of *G* (see Figure 1). Then, *u*_1_ and *u*_2_ are contained in the same color class for any 3-coloring of *G*. Let *G*′ be the graph obtained from *G* by identifying *u*_1_ and *u*_2_. Then, *G* is 3-colorable if and only if *G*′ is 3-colorable.

The following lemmas are useful for our proofs.

**Lemma 2 (see [5]).**

Every simple planar graph *G* contains (i) a vertex of degree at most 4 or (ii) a bad vertex.

**Lemma 3 (see [5]).**

Let *G* be a plane graph with a 4-coloring *c*′, and let *f* be a face of size at least four. Take four vertices *x*_1_, *x*_2_, *x*_3_, and *x*_4_ (along clockwise order) in *f*. Then, *G* also has a 4-coloring such that at most three colors are used for *x*_1_, *x*_2_, *x*_3_, and *x*_4_. Moreover, given the graph *G* and the coloring *c*′, we can find such a 4-coloring of *G* in *O*(*n*) time, where *n*=|*G*|.

**Lemma 4 (see [6]).**

If *v* is a vertex of planar graph *G* and *G*[*N*[*v*]] is {claw, *K*_4_}-free, then *d*(*v*) ≤ 5 and *G*[*N*[*v*]] is a 5-wheel if *d*(*v*)=5.

**Figure 2:**
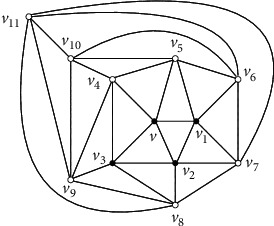
The icosahedron.

The *icosahedron* is the 5-regular planar graph in Figure 2. In order to give our algorithm, we first give a well known fact about planar graphs.

**Observation 5 .**

The icosahedron is the unique 5-regular planar graph *G* such that, for every vertex *v* ∈ *V*(*G*), the induced subgraph *G*[*N*[*v*]] is a 5-wheel.

Further, we can prove the following theorem.

**Theorem 6 .**

Let *G* be a claw-free planar graph. If *G* is not the icosahedron, then *G* has a vertex of degree at most four.

**Proof**

Let *G*=(*V*, *E*) be a claw-free planar graph. If *G* has no vertex of degree at most four, then *δ*(*G*) ≥ 5. In the following, we just prove that *G* is the icosahedron. If *G* has no *K*_4_, by Lemma 4, every vertex *v* of *G* induces a 5-wheel of *G*. So, by Observation 5, *G* is the icosahedron. If not, let [*x*_1_*x*_2_*x*_3_*x*_4_] be a *K*_4_ of *G* and assume that *x*_1_ is inside the triangle [*x*_2_*x*_3_*x*_4_] in the embedding of *G* on the plane. Without losing of the generality, we may assume that there is no other *K*_4_ inside the triangle [*x*_2_*x*_3_*x*_4_]. Suppose not, we can continue to find another *K*_4_ inside the triangle [*x*_2_*x*_3_*x*_4_] and consider the inner situation in this *K*_4_ until finding such a triangle since *G* is a finite graph. Note that *d*(*x*_1_) ≥ 5. Assume that some neighbors of *x*_1_ are inside the triangle [*x*_1_*x*_2_*x*_3_]. Let *G*^*∗*^ be the plane graph induced by the vertices on and inside [*x*_1_*x*_2_*x*_3_]. We have the following claim.

**Claim 1 .**

For every vertex *x* inside [*x*_1_*x*_2_*x*_3_], *G*[*N*[*x*]]=*G*^*∗*^[*N*[*x*]] and *G*[*N*[*x*]] is isomorphic to a 5-wheel by Lemma 4 and the assumption that there is no *K*_4_ in *G*^*∗*^.

**Figure 3:**
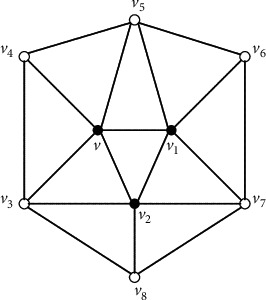
*G*[*N*[{*v*, *v*_1_, *v*_2_}]].

**Figure 4:**
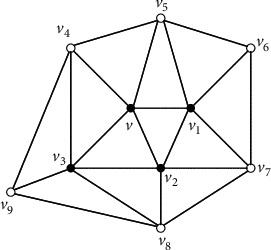
*G*[*N*[{*v*, *v*_1_, *v*_2_, *v*_3_}]].

**Figure 5:**
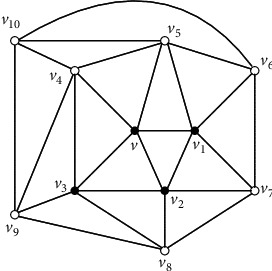
*G*[*N*[{*v*, *v*_1_, *v*_2_, *v*_3_, *v*_4_, *v*_5_}]].

Take any vertex *v* in *V*(*G*^*∗*^) − {*x*_1_, *x*_2_, *x*_3_}. *G*[*N*[*v*]] is isomorphic to a 5-wheel by Claim 1. Let *C*_*v*_=*v*_1_*v*_2_*v*_3_*v*_4_*v*_5_*v*_1_ be the cycle of 5-wheel *G*[*N*[*v*]]. Note that at least three neighbors of *v* are inside the triangle [*x*_1_*x*_2_*x*_3_] since it is impossible that three neighbors of *v* are the vertices of the triangle [*x*_1_*x*_2_*x*_3_]. Without losing of the generality, we assume that *v*_1_, *v*_2_, and *v*_3_ are inside [*x*_1_*x*_2_*x*_3_]. Then, *G*[*N*[*v*_1_]], *G*[*N*[*v*_2_]], and *G*[*N*[*v*_3_]] are isomorphic to the 5-wheel by Claim 1. Let *C*_*v*_1__=*v*_2_*vv*_5_*v*_6_*v*_7_*v*_2_ be the cycle of 5-wheel *G*[*N*[*v*_1_]] and *C*_*v*_2__=*v*_3_*vv*_1_*v*_7_*v*_8_*v*_3_ be the cycle of 5-wheel *G*[*N*[*v*_2_]] (see Figure 3). Further, let *C*_*v*_3__=*v*_4_*vv*_2_*v*_8_*v*_9_*v*_4_ be the cycle of 5-wheel *G*[*N*[*v*_3_]] (see Figure 4). Note that *v*_4_ is not adjacent to *v*_8_ in Figure 4, without losing of the generality, assume that *v*_4_ is inside [*x*_1_*x*_2_*x*_3_]. Then, *G*[*N*[*v*_4_]] is a 5-wheel, and we claim that neither *v*_6_ nor *v*_7_ is the fifth neighbor of *v*_4_. If *v*_7_ is the fifth neighbor of *v*_4_, by the fact that *G*[*N*[*v*_4_]] is a 5-wheel, *v*_5_ is also adjacent to *v*_7_, a contradiction to the fact that *G*[*N*[*v*_1_]] is a 5-wheel. If *v*_6_ is the fifth neighbor of *v*_4_, by the fact that *G*[*N*[*v*_4_]] is a 5-wheel, *v*_9_ is also adjacent to *v*_6_. Then, no matter how we draw the two edges *v*_4_*v*_6_ and *v*_9_*v*_6_ in the plane, we can get a vertex of degree four by the claw-freeness, a contradiction. So, let *C*_*v*_4__=*v*_5_*vv*_3_*v*_9_*v*_10_*v*_5_ be the cycle of 5-wheel *G*[*N*[*v*_4_]]. By the claw-freeness, *v*_10_ is adjacent to *v*_6_. We claim that *v*_5_ is also inside [*x*_1_*x*_2_*x*_3_], and thus *G*[*N*[*v*_5_]] is a 5-wheel (see Figure 5). Suppose not, if *v*_5_=*x*_1_, {*x*_4_, *v*_5_(*x*_1_), *v*_4_, *v*_1_} would induce a claw, a contradiction. Consider the vertices in {*v*_6_, *v*_7_, *v*_8_, *v*_9_, *v*_10_} in Figure 5. Without losing of the generality, assume that *v*_10_ is inside [*x*_1_*x*_2_*x*_3_]. Then, *d*_*G*^*∗*^_(*v*_10_)=5, and *G*[*N*[*v*_10_]] is a 5-wheel. We can easily see that neither *v*_7_ nor *v*_8_ is the fifth neighbor of *v*_10_. So, let *C*_*v*_10__=*v*_9_*v*_4_*v*_5_*v*_6_*v*_11_*v*_9_ be the cycle of 5-wheel *G*[*N*[*v*_10_]]. Hence, {*v*_11_*v*_6_, *v*_11_*v*_9_} ⊂ *E*(*G*^*∗*^) by the claw-freeness and both *v*_6_ and *v*_9_ are inside [*x*_1_*x*_2_*x*_3_]. Further, we have that {*v*_11_*v*_7_, *v*_11_*v*_8_} ⊂ *E*(*G*^*∗*^) by the claw-freeness (see Figure 2). Further, by the claw-freeness, no one in {*v*_7_, *v*_8_, *v*_11_} is in the set {*x*_1_, *x*_2_, *x*_3_}. Suppose not, if *v*_11_=*x*_1_, {*x*_4_, *v*_11_(*x*_1_), *v*_6_, *v*_9_} would induce a claw, a contradiction. Then, all the vertices inside the triangle [*x*_1_*x*_2_*x*_3_] induce the icosahedron (see Figure 2), still a contradiction to the assumption that some neighbor of *x*_1_ is inside the triangle [*x*_1_*x*_2_*x*_3_].

Immediately, we have the following corollary.

**Corollary 7 .**

The icosahedron is the unique 5-regular planar graph such that every vertex is a bad vertex of Type I.

**Proof**

Let *G*=(*V*, *E*) be a 5-regular planar graph such that every vertex is a bad vertex of Type I. Then, *G* is also a claw-free planar graph. By Theorem 6, *G* is the icosahedron.

Based on Observation 1, Lemma 2, Lemma 3, Corollary 7, and Theorem 6, we design an algorithm as follows.

**Theorem 8 .**

Given a planar graph *G*, Algorithm 1 is a linear-time algorithm to output either a 4-coloring of *G* or the decision that *G* is not 3-colorable. In addition, if *G* is a 3-colorable planar graph or a planar graph with maximum degree at most five or a claw-free planar graph, Algorithm 1 gives a 4-coloring of *G*.

**Proof**

Based on Lemmas 2 and 3 and the fact that identifying *u*_1_ and *u*_2_ in step 2 keeps the 3-colorability of *G*, we can say that Algorithm 1 outputs either a 4-coloring of *G* or the decision that *G* is not 3-colorable. Now, we prove that Algorithm 1 is a linear-time algorithm by induction on the order of *G*. Assume that it holds when the order of *G* is less than *n*. Then, let |*G*|=*n*. In step 1, we can find such a partition in at most *O*(*n*) time. In step 2, we can give a 4-coloring of *G* directly in constant time if *G* is the icosahedron or has at most four vertices. If not, we can construct a graph *G*′ in constant time at most. Note that |*G*′|=*n* − 1. By induction, *A*(*G*′) needs *O*(*n* − 1) time which is the total time of all repeated steps from 1 to 5 in *A*(*G*′). In step 3, it needs only the constant time by deciding the result of *A*(*G*′). In step 4, we can give a 4-coloring of *G* from the 4-coloring of *G*′ in *O*(*n*) time by Lemma 3. In step 5, it needs only the constant time. Thus, the total time is also *O*(*n*).

If *G* is a 3-colorable planar graph, we are sure to get a 4-coloring of *G* by Observation 1 and Lemmas 2 and 3. If *G* is a planar graph with maximum degree at most five, we just consider the case that a vertex of degree greater than five occurs in *G*′ by identifying *u*_1_ and *u*_2_ of *G* in step 2. When a vertex of degree greater than five occurs in *G*′, then *v* becomes a vertex of degree four of *G*′ (see Figure 1). According to our algorithm, we first take a vertex *v* of degree at most 4 in step 2 when we carry out *A*(*G*′). Hence, after several steps, the vertex of degree greater than five will disappear. So, the final *G*′ will be the icosahedron or a graph with at most four vertices by Corollary 7, and we get a 4-coloring of *G* at last. If *G* is a claw-free planar graph, we can get a 4-coloring of *G* by Lemma 3 and Theorem 6.

## Figures and Tables

**Algorithm 1:**
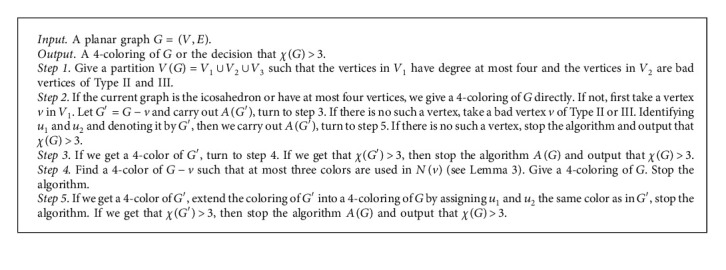
An algorithm of coloring planar graphs.

## Data Availability

Our result is supported by the rigorous proofs in the submitted paper.
